# The importance of pharmacokinetics and pharmacodynamics in antimicrobial drug development and their influence on the success of agents developed to combat resistant gram negative pathogens: A review

**DOI:** 10.3389/fphar.2022.888079

**Published:** 2022-07-25

**Authors:** Mary E. Palmer, Lauren J. Andrews, Taylor C. Abbey, Ashley E. Dahlquist, Eric Wenzler

**Affiliations:** College of Pharmacy, University of Illinois Chicago, Chicago, IL, United States

**Keywords:** pharmacokinetics, pharmacodynamics, drug development, antibiotic resistance, antimicrobial

## Abstract

A deep understanding of an antimicrobial’s critical pharmacokinetic and pharmacodynamic properties is crucial towards optimizing its use in patients and bolstering the drug development program. With the growing threat of antimicrobial resistance and decline in antimicrobial development, the advancement of complex and rigorous pharmacokinetic and pharmacodynamic studies over a short time span has renewed confidence in the value of pharmacokinetic and pharmacodynamic studies and allowed it to become fundamental component of a robust drug development program with high chances of successful approval. In addition, recent guidance by various regulatory bodies have reinforced that a strong and dedicated focus on pharmacokinetics and pharmacodynamics throughout research and development lead to the use of an optimized dosing regimen in Phase 3 trials, improving the probability of drug approval. The objective of this review is to demonstrate the importance of pharmacokinetic and pharmacodynamic studies in the drug development decision-making process by highlighting the developments in pharmacokinetic and pharmacodynamic methods and discuss the role of pharmacokinetic and pharmacodynamic studies in antimicrobial successes and failures.

## Introduction

The World Health Organization has declared antimicrobial resistance as one of the top ten global public health threats facing humanity (World Health Organization, 2019). According to the 2019 Centers for Disease Control and Prevention (CDC) Antibiotic Resistance Threat Report, over 2.8 million antibiotic-resistant infections occur each year in the United States resulting in at least 35,000 deaths (CDC, 2019). Importantly, 10 of the 18 threats named in the CDC report are Gram-negative pathogens, with 50% of the aforementioned 2.8 million infections being caused by extended-spectrum β-lactamase (ESBL)-producing Enterobacterales. Unfortunately, while the prevalence of and morbidity and mortality associated with these pathogens continues to increase, the number of new antimicrobial compounds being developed to treat these infections lags. Since 2014, only nine antibiotics have been approved that target resistant Gram-negative pathogens (Table 1) and 78% of major pharmaceutical companies have scaled back or cut antibiotic research due to developmental challenges (PEW, 2021). Many of these challenges include monetary costs, time, feasibility of clinical trials, regulatory barriers, and low return on investment (Luepke and Mohr, 2017; Bhavnani et al., 2020; Safir et al., 2020). As resistance has increased and drug development slowed, pharmacokinetic (PK) and pharmacodynamic (PD) studies have become even more important as a means of optimizing and preserving the agents currently available. Pharmacokinetics is the study of the time course of drug absorption, distribution, metabolism, and excretion, while PD is the relationship between drug concentrations at sites of action and resulting effects. Antimicrobials are unique in that they are the only pharmacologic agents which exert their activity on another living organism. The integration of PK and PD helps define the dose-exposure-response relationship which is a crucial step in maximizing the efficacy and minimizing the toxicity of a given agent, ultimately leading to improved patient outcomes and prolonging the lifespan of the drug (Drusano, 2004). The introduction of rigorous PK/PD methods and an improved understanding of the importance of dose optimization has revolutionized the antibacterial development process by minimizing costs, maximizing efficacy and resistance prevention, minimizing toxicity, and helping to avoid failures in clinical studies by preventing suboptimal study designs in Phase II and III trials (Bhavnani et al., 2020). These advanced PK/PD methods such as population PK modeling and Monte Carlo simulation also allow for antimicrobial dosing schemes to be optimized post-marketing and adapted to target patient populations, such as the critically ill. Given the rapid and continued evolution of the science and art of antimicrobial PK/PD and the dynamic nature of the regulatory environment in the United States, herein we sought to emphasize the importance of PK/PD studies for decision making in the antimicrobial drug development process, highlight developments in PK/PD methods, and discuss the role of PK/PD in notable drug successes and failures.

**TABLE 1:** Antibiotics currently in global clinical development. Abbreviations: PBP, penicillin-binding protein; ESBL, extended-spectrum beta-lactamase; CRE, carbapenem-resistant Enterobacterales; CRPA, carbapenem-resistant *P. aeruginosa*; CRAB, carbapenem-resistant *A. baumannii*.

Drug name	Development phase	Company	Drug class	Novel drug class?	Target	Novel target?	Expected activity against eskape pathogens?	Expected activity against CDC urgent or WHO critical threat pathogen?
Sulopenem/sulopenem etzadroxil- probenecid	New drug application submitted (U.S. FDA)	Iterum Therapeutics PLC	β-lactam (thiopenem)	No	PBP	No	Yes: *K. pneumoniae*, *Enterobacter* spp.	Yes: ESBL, drug-resistant *N. gonorrhoeae*
Benapenem	Phase 3	Sihuan Pharmaceutical Co. Ltd	Carbapenem	No	PBP	No	Yes: *K. pneumoniae, Enterobacter* spp.	Yes: ESBL
Cefepime + taniborbactam	Phase 3	Venatorx Pharmaceuticals Inc./Global Antibiotic Research and Development Partnership (GARDP) (Everest Medicines II Ltd. Licensee)	β-lactam (cephalosporin) + β-lactamase inhibitor (cyclic boronate)	No	PBP + β-lactamase	No	Yes: *K. pneumoniae*, *P. aeruginosa*, *Enterobacter* spp. Possibly: *S. aureus*	Yes: CRE, CRPA
EMROK/EMROK O	Phase 3	Wockhardt Ltd	Fluoroquinolone	No	Bacterial type II topoisomerase	No	Yes: *S. aureus*	No
Exblifep (cefepime + enmetazobactam)	Phase 3	Allecra Therapeutics GmbH	β-lactam (cephalosporin) + β-lactamase inhibitor (penicillanic acid sulfone)	No	PBP + β-lactamase	No	Yes: *K. pneumoniae*, *Enterobacter* spp.	Yes: ESBL
Gepotidacin (GSK2140944)	Phase 3	GlaxoSmithKline PLC	Triazaacenaphthylene	Yes	Bacterial type II topoisomerase (novel A subunit site)	Yes	Yes: *S. aureus*	Yes: Drug-resistant *N. gonorrhoeae*
								Possibly: ESBL
Sulbactam + durlobactam	Phase 3	Entasis Therapeutics Inc	β-lactam (sulbactam)+ β-lactamase inhibitor (diazabicyclooctane)	No	PBP + β-lactamase	No	Yes: *A. baumannii*	Yes: CRAB
Tebipenem/tebipenem pivoxil hydrobromide	Phase 3	Spero Therapeutics Inc	β-lactam (carbapenem)	No	PBP	No	Yes: *K. pneumoniae*, *Enterobacter* spp.	Yes: ESBL
								Possibly: *C. difficile*
WCK 5222 (cefepime + zidebactam)	Phase 3*	Wockhardt Ltd	β-lactam (cephalosporin) + β-lactamase inhibitor (diazabicyclooctane)	No	PBP + β-lactamase	No	Yes: *K. pneumoniae*, *Enterobacter* spp.	Yes: CRE
							Possibly: *S. aureus*, *P. aeruginosa*	Possibly: CRPA
Zevtera (ceftobiprole)	Phase 3	Basilea Pharmaceutica International Ltd	β-lactam (cephalosporin)	No	PBP	No	Yes: *S. aureus*, *K. pneumoniae*, *Enterobacter* spp.	No
							Possibly: *P. aeruginosa*	

## PK/PD in drug development

For many years, antibiotics were readily available without having to prove efficacy or safety in rigorous clinical trials (Luepke and Mohr, 2017). In response, legislation began to be introduced over time to address these gaps in the drug development process. Then in 2002, the United States Food and Drug Administration (FDA), European Medicines Agency (EMA), and other regulatory bodies proposed strict guidance for the appropriate conduct of Phase III trials for antibacterial agents. The requirements, in part, resulted in a mass exodus of large pharmaceutical companies out of the antimicrobial drug development arena (Bhavnani et al., 2020). While smaller companies attempted to fill and capitalize on this void, their comparatively limited resources made it nearly impossible to carry a compound from discovery and interest in new antibiotic discovery faded (Bhavnani et al., 2020). Recognizing the enormous public health consequences stemming from a lack of antimicrobial innovation, there has been a resurgence of interest and financial and regulatory support towards the research and development of new antibiotics over the last decade. The antibiotic market became incentivized by government agencies and large private-public partnerships such as BARDA and CARB-X which started to push more antimicrobials through development and into commercial availability (Luepke and Mohr, 2017). Along with this renewed interest in antimicrobial drug discover came the recognition of the importance of pharmacology in translational medicine, especially as it applied to combating resistant Gram-negative pathogens given the difficulties in completing large, rigorous clinical trials in this area (Rizk et al., 2019). Terms such as pharmacometrics and model-based drug development quickly became commonplace and the science of rigor of antimicrobial PK/PD advanced rapidly. Pharmacometrics utilizes PK and PD data to generate models characterizing aspects of drug efficacy such as disease progression, therapy adherence, and bacterial growth to provide guidance in trial design, efficacy comparisons, dose optimization, and decisions regarding care in specific patient population (Gallo, 2010). Advances in the field of pharmacometrics played a crucial role in solidifying the place of PK/PD in the drug development process and repeatedly demonstrating its immense value by reducing costs and shortening the drug approval timeline (Trivedi et al., 2013).

Eventually, regulatory agencies such as the FDA also began to take notice of the impact of PK/PD and revised their guidance for industry to encourage sponsors to establish a strong foundation in PK/PD and develop a deep understanding of how to optimize the use of their drug long before entering Phase 3 clinical trials. Before this shift in focus on PK/PD studies, PK and PD were often studied independent of each other with very little regulatory guidance on how these studies should be conducted. The FDA mandated studies to provide PK characteristics but not establish the relationship between these characteristics and PD properties (Gallo, 2010). As a result, drug distribution and understanding drug disposition in various tissue targets were not the focus of many pharmaceutical companies in drug development. Any medications deemed “ineffective” may have failed due to PK/PD issues but the lack of insight and knowledge in this area hindered opportunities to improve and learn from drug failures (Gallo, 2010). In the current era of antimicrobial development, PK/PD data are integrated into the process early on to help understand drug mechanisms of action, select optimal lead compounds, support dose selection, minimize animal usage, shorten the development time, estimate the therapeutic index, and provide more certainty ahead of larger, time-consuming and costly clinical trials (Tuntland et al., 2014; Rizk et al., 2019). Regulatory agencies outside the United States have been even further ahead in their commitment to the importance of PK/PD in antibiotic development. In the 2000s, The European Agency for the Evaluation of Medicinal Products’ Committee for Proprietary Medicinal Products (CPMP) created a document titled “Points to Consider on Pharmacokinetics and Pharmacodynamics in the Development of Antibacterial Medicinal Products” (European Medicines Agency, 2009). This document acknowledged that “there seems to be sufficient evidence to support a recommendation that the PK/PD relationship for an antibacterial medicinal product should be investigated during the drug development program.”

Pursuant to the urgent need for antimicrobial agents for resistant Gram-negative infections, regulatory agencies have placed more emphasis on PK/PD data to help antimicrobials gain market approval. In 2016, the FDA conducted a workshop to address challenges and place emphasis on the need for these specific antimicrobial agents. In response to this workshop, the Infectious Diseases Society of America (IDSA) published a white paper that summarized various approaches for clinical trial design and data packages, including PK/PD data, in support of developing newer antimicrobial agents (Bhavnani and Rex, 2017). As the field of pharmacometrics advanced even further, the EMA created an updated document titled “Guideline on the use of pharmacokinetics and pharmacodynamics in the development of antibacterial medicinal products” in 2015 (European Medicines Agency, 2016). This effectively replaced the CPMP guidance document and focused on the use of PK/PD analyses to identify potentially efficacious dosing regimens. The EMA Guideline addresses the following: microbiological data that should be accumulated to support PK/PD analyses, including descriptions of MIC distributions and time-kill studies; identification of PK/PD indices and PK/PD targets from pre-clinical data using *in vitro* and/or *in vivo* PD models; clinical PK data needed across various diseases states, age groups, and therapies to support PK/PD analyses at various stages of clinical development; determination of the probability of target attainment (PTA) using simulations to support dose regimen selection; evaluation of clinical exposure-response relationships using data collected during clinical studies that assess clinical and microbiological patient outcomes; identification of beta-lactamase inhibitor dose regimens; and the extent to which the results of PK/PD analyses may support or replace clinical data (European Medicines Agency, 2016). The FDA has followed suite by establishing a Division of Pharmacometrics that has helped to provide guidance documents on end-of-phase 2A meetings, population pharmacokinetics, exposure-response relationships encompassing study design, data analysis, and regulatory applications, E4 dose-response information to support drug registration, and various documents on special populations (US Department of Health and Human Services and Food and Drug Administration, 2021). In 2018, the FDA issued the Physiologically Based PK (PBPK) Analyses Format and Content program to provide recommendations for sponsors and applicants on the format and content of PBPK analyses to supplement new drug applications. This includes reviewing the adequacy of submitted PBPK models in their ability to support intended purposes at different stages of drug development, facilitating investigational new drug (IND) and new drug application (NDA) review process through *de novo* analyses, supporting regulatory policy via scientific research and maintenance of a PBPK knowledgebase, harmonizing regulatory recommendations on the use of PBPK with non-US regulatory body, and reaching out to the scientific community to advance the science of PBPK (US Department of Health and Human Services and Food and Drug Administration, 2021). The involvement and cooperation of regulatory agencies within and outside the United States has continued to propel the science of antimicrobial PK/PD forward thereby streamlining the development process and ultimately creating safer and more efficacious agents capable of generating greater post-approval success.

## Evolution OF PK/PD

As previously mentioned, the 2015 EMA guideline on the use of PK and PD in the development of antibacterial medicinal products was created in large part as a response to the expansion and improvement of PK/PD models. Current PK/PD model systems are a far cry from the first notable use of PK/PD analysis was in the 1940s–1950s by Dr. Harry Eagle via investigations of time-dependent killing of penicillin (Labreche et al., 2015; Bhavnani and Rex, 2017). In the 1970s, Dr. William Craig used *ex vivo* and *in vivo* models to help classify antimicrobials as time- or concentration-dependent. He further demonstrated the significance of protein binding, characterized post-antibiotic effects, and first utilized dose-fractionation studies to identify PK/PD indexes associated with treatment efficacy (Labreche et al., 2015; Bhavnani and Rex, 2017). Through the 1990s and 2000s, clinical data from infected human subjects used to characterize PK/PD relationships for efficacy of aminoglycosides, fluoroquinolones, and beta-lactams reinforced the data from animal models, highlighting the exciting potential of pre-clinical PK/PD studies (Labreche et al., 2015; Bhavnani and Rex, 2017). In the late 1990s, Monte Carlo simulations began to be used to determine the antimicrobial dose selection window and PTA through the use of inter-patient PK variability on target exposures. Since then, the widespread use of these simulations in combination with non-clinical PK/PD targets, population PK models, and *in vitro* surveillance data have been used to evaluate dosing regimens and criteria for *in vitro* susceptibility testing and clinical breakpoints during drug development and approval.

Throughout this maturation process numerous important pillars instrumental to conducting robust and reliable PK/PD studies have emerged and served to lay the foundation for future technological and scientific advances in this field. One of these tenants was the importance of studying an agent across multiple different pre-clinical PK/PD model systems and collating and translating this data into more informed clinical studies. During preclinical evaluations, animal and *in vitro* models have been vital to helping to determine indications and dosing regimens, decreasing sample sizes needed in clinical trials, and overall increasing the success rates and accelerating drug development (Rodriguez-Gascon et al., 2021). *In vitro* models are less cost- and resource-intensive and permit investigations of longer durations and higher bacteria inoculum than animal models, which is especially helpful when detecting resistance mutations and subpopulations. Dynamic one-compartment and two-compartment PK/PD studies allow for the precise simulation of human concentration-time profiles. A downside to *in vitro* models is a lack of immune components, making it difficult to extrapolate results to immunocompetent hosts (Rodriguez-Gascon et al., 2021). Conversely, animal models help to simulate the pathophysiology of infections in human patients and are useful in examining efficacy at specific body sites including endocarditis, the genitourinary tract, and wounds. Although these models are now required for entering first-in-human studies and to obtain market approval, they are hampered by the difficulty in translating PK properties between mammals and humans. Even with the use of these models in drug development, it is still difficult to predict and interpret clinical outcome data from these pre-clinical models. The introduction of time-kill analyses (MIC-based or mechanism-based) has helped describe bacterial growth and death rates, drug effects, and resistance emergence within a population. These models have become more complex over the years and can even help to evaluate combinations of antibiotics. The killing effect of aztreonam-avibactam on drug susceptible and resistant bacteria is an example of a mechanistic time-kill model (Rodriguez-Gascon et al., 2021). The development of population PK models has helped to predict human exposures to antimicrobial agents and to explore exposure-response relationships. These models help to bridge the gap between preclinical data and clinical trials. When choosing a dosing regimen, teetering the line between toxicity and efficacy may be challenging. Population PK models can help determine the dose by placing the maximum number of individuals within the therapeutic window and dosing regimens can be tailored to specific patient groups such as those with renal or hepatic sufficiency, obesity, or critically ill (Rodriguez-Gascon et al., 2021). Additionally, population PK helps identify demographical, pathophysiological, and therapeutic information in addition to any other parameters that could be responsible for differences in achieved drug concentrations.

Population PK analyses in conjunction with Monte Carlo simulations have revolutionized the ability to set and revise antimicrobial susceptibility breakpoints which are the linchpin necessary to translate and apply pre-clinical PK/PD information into the clinical arena and inform optimal dosing based on patient-specific factors and the pathogen MIC. These models have been especially useful to the Clinical and Laboratory Standards Institute (CLSI) and European Committee on Antimicrobial Susceptibility Testing (EUCAST) when setting and revising breakpoints for multidrug resistant Gram-negative pathogens (Rodriguez-Gascon et al., 2021). Monte Carlo simulations started by using basic PK data and have since evolved and improved to using a validated population PK model built from the infected target patient population, a variability model, covariate model, and a PD model where the interrelationship of the PK and PD parameters has been studied. Different PK parameters can be used in Monte Carlo simulations in varying patient populations, even after market approval, such as cystic fibrosis, ICU patients, obesity, pediatrics, and geriatrics leading to differences in derived breakpoints, and these populations may need higher antibiotic dosing than healthy human patients. CLSI breakpoints may not always take these patient populations into account when setting breakpoints, so seeking out studies using Monte Carlo simulations and comparing CLSI and EUCAST breakpoints can be especially helpful in ensuring dose optimization (Rodriguez-Gascon et al., 2021) Lastly, PK/PD tools have been established for surveillance of antibacterial activity and prediction of drug resistance. Dosing regimens can be selected to minimize the emergence of resistant mutations. These newer PK/PD models have helped to further tailor antibiotic dosing to fit the needs of special populations and lead to the successful approval of newer antibiotics.

## Notable failures and successes

**FIGURE 1:**
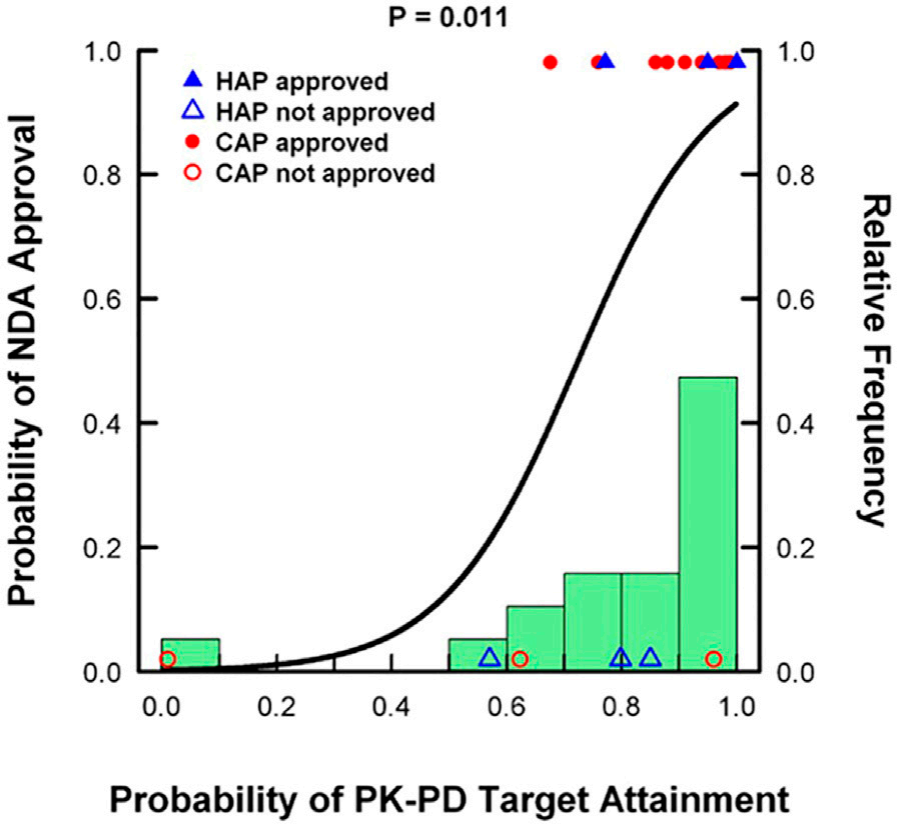
Analysis of 19 antibacterial drug approval candidates from 1996 to 2011 demonstrating the correlation between PK/PD PTA and the probability of regulatory approval. Adapted from: Bulik CC, Bhavnani SM, Hammel J, Forrest A, Dudley MN, Ellis-Grosse EJ, Drusano GL, Ambrose, PG. Relationship between regulatory approval and pharmacokinetic-pharmacodynamic target attainment: Focus on community- and hospital-acquired pneumonia [Abstract A-295]. 53rd Interscience Conference on Antimicrobial Agents and Chemotherapy. Denver, CO. September 10–13, 2013.

Optimizing antimicrobial pharmacometrics helps to improve the chances of success in both the drug development and patient care phases while being cost and time effective. As shown in Figure 1, there is a strong, significant positive correlation between designing a dosing regimen that can achieve the necessary PK/PD PTA targets and successfully earning regulatory NDA approval. Despite this strong correlation, the drug development process is complex and challenging and the ultimate success or failure of a compound hinges on a multitude of factors. These include understanding the primary target pathogen(s), determining the appropriate PK/PD indices and susceptibility breakpoints, developing optimal dosing regimens for both efficacy and safety, and optimizing study design in clinical development and post-marketing evaluations (Trivedi et al., 2013). In order to help hone antimicrobial dosing, combining MIC as a PD parameter in combination with PK exposure defines PK/PD indices related to target exposure, microbial sensitivity, and PK/PD breakpoints. The main three PK/PD indices used to classify the killing activity of antimicrobials are time T of microorganisms to plasma concentrations exceeding their MIC (T > MIC), ratio of peak plasma concentration to MIC (Cmax/MIC), and ratio of area under plasma concentration curve to MIC (AUC/MIC) (Trivedi et al., 2013). Understanding this aspect of the antimicrobial is the first-step in fully understanding how to choose an effective dosage regimen. Additionally, our greater understanding of PK/PD has helped solidify concepts crucial to the optimal use of antibiotics in patients with multidrug resistant Gram-negative infections, such as the importance of adequate target site concentrations relative to the pathogen MIC (Trivedi et al., 2013). The discipline of antimicrobial PK/PD has clearly demonstrated its value in the regulatory and drug approval process and as an instrumental tool in ensuring that the safest and most efficacious drugs and dosing regimens reach our patients. Despite this, the emphasis placed on establishing a robust PK/PD program during the drug development process varies across antibiotic manufacturers, pharmaceutical companies, regulatory agencies, and clinicians. Exploring the potential underlying PK/PD explanations for notable antimicrobial drug development successes and failures can continue to improve our knowledge and the efficiency and timeliness of the approval period.

### Meropenem-vaborbactam

Vaborbactam is a cyclic boronic-acid β-lactamase inhibitor that was designed specifically to have potent activity against the KPC enzyme most often found in carbapenem-resistant *K. pneumoniae.* Meanwhile the broad spectrum *in vitro* activity, well-established safety profile, efficacy against serious Gram-negative infections, and comparable PK made meropenem the ideal partner agent. Before establishing meropenem and vaborbactam compatibility, vaborbactam PK was assessed via a multiple-dose PK study in rats and demonstrated linear PK and dose proportionality, concentration-time profiles displayed a high Cmax and AUC, a low Vd, and short half-life (Wenzler and Scoble, 2020) Additionally, toxicology studies in dogs concluded that multiple doses of vaborbactam up to the human equivalent of 10 g/day did not reveal any dose-limiting toxicities (Wenzler and Scoble, 2020). These preclinical studies helped to build up vaborbactam’s profile as an excellent match for a beta-lactam antibiotic, especially meropenem. To establish comparability in PK with meropenem, multiple single-center, Phase 1 studies were conducted (Griffith et al., 2016; Rubino et al., 2018a). The first included 80 healthy subjects in a multiple-ascending dose study. Six subjects were randomized to receive vaborbactam ranging from 250 to 2000 mg or placebo administered as a 3-h infusion across ten cohorts, with the first six cohorts receiving doses from 250 to 1,500 mg and the remaining four cohorts receiving doses ranging from 250 to 2000 mg q8h for 7 days. The exposure of vaborbactam based on Cmax and AUC increased proportionally to dose after both single and repeated doses, confirming linear PK throughout the dosing range. Additionally, PK parameters and renal clearance remained unchanged following repeated dosing, which suggested a lack of accumulation in plasma at an every 8-h dosing interval (Griffith et al., 2016) The next single-center phase I study assessed PK of vaborbactam alone and in combination with meropenem in 80 healthy subjects. Single and multiple doses were given as a 3-h infusion across only five dosing cohorts this time. Meropenem was administered as 1 and 2 g doses in combination with vaborbactam doses of 250 mg, 1 g, 1.5 g and 2 g. Meropenem and vaborbactam demonstrated linear PK and dose-proportional increases in AUC across the dosing ranges, the geometric mean half-life of meropenem and vaborbactam was 0.9–1.3 h and 1.1–1.9 h respectively, further confirming the comparability of their PK profiles. Doses of up to a total 4 g (2 g meropenem +2 g vaborbactam) q8h were well-tolerated as well (Rubino et al., 2018a). Both meropenem and vaborbactam are predominantly renally eliminated, and one potential issue is the faster clearance of vaborbactam compared to meropenem especially in populations with renal impairment. This would potentially lead to a decreased efficacy of the meropenem component of the combination antibiotic as the vaborbactam component is preventing meropenem hydrolysis via beta-lactamases, especially KPC-producing bacteria (Wenzler and Scoble, 2020) In an ideal world, both would be equally eliminated in this population. A phase I, multicenter study of 41 patients with chronic renal impairment was conducted to assess clearance. A single fixed dose of 1 g of meropenem plus 1 g vaborbactam as a 3-h infusion was given to all patients except for those on hemodialysis, who received a dose both on and off dialysis. Overall exposure to both components increased as renal function deteriorated. The average AUC_0-∞_ for meropenem increased approximately 4.5-fold from 87.1 mg/l in subjects with healthy and normal renal function to 397 mg/l in those with severe renal impairment. For vaborbactam, exposure increased almost 8-fold from 99.4 mg/l to 781 mg/l (Rubino et al., 2018b). Similar findings were also noted in the hemodialysis population. No additional adverse events were noted in this study. This helped to establish meropenem-vaborbactam dosing in patients with renal impairment and determine that a fixed dose of meropenem-vaborbactam was appropriate. A total dose of 4 g IV q8h as a 3-h infusion was ultimately chosen to be used in phase 3 clinical trials to address increased resistance in Gram-negative bacteria, especially CRE. This dose provided bactericidal activity against meropenem-resistant Enterobacterales isolates with a 6-log_10_ CFU/ml decrease and a high threshold for resistance development (Wenzler and Scoble, 2020). Probabilities of target attainment for Enterobacteriaceae species ranged from 94.4 to 100% at a MIC value of 8 μg/ml.The resume built from these preclinical and phase I studies helped set up the success in phase 3 clinical trials and ultimate regulatory approval in complicated urinary tract infections (cUTI) and acute pyelonephritis.

Before these phase 3 clinical trials were conducted, researchers first sought to determine if this total dose of 4 g IV q8h as a 3-h infusion would penetrate the areas to be studied. This was extremely important to the success of TANGO I and TANGO II. Prior to TANGO I, which looked at meropenem/vaborbactam versus piperacillin/tazobactam in patients with cUTI, a murine model of pyelonephritis was performed to evaluate the dosing regimen. Mice were administered meropenem doses at 100 or 300 mg/kg every 2 h over 24 h which produced an exposure equivalent of 1 or 2 g of meropenem every 8 h by 3-h infusion in humans and administered vaborbactam at 25 or 50 mg/kg every 2 h for 24 h which was equivalent to 1 or 2 g of vaborbactam every 8 h by 3-h infusion in humans. A dose of meropenem 300 mg/kg alone reduced the bacterial load in the kidneys by an average of 1.51 log10 CFU while the combination of meropenem 300 mg/kg plus vaborbactam 50 mg/kg resulted in bacterial kidney titers that were 2.89 log10 CFU lower than the controls and 1.38 log10 CFU lower than meropenem alone. Mice that received meropenem 300 mg/kg plus vaborbactam 50 mg/kg had a larger reduction in bacterial load compared to meropenem 100 mg/kg plus 50 mg/kg or 25 mg/kg of vaborbactam. (Weiss et al., 2018). The meropenem 300 mg/kg plus vaborbactam 50 mg/kg dosing regimen is equivalent to the meropenem 4 g dosing regimen used in humans. In TANGO I, meropenem/vaborbactam resulted in a composite outcome of complete resolution or improvement of symptoms along with microbial eradication that met noninferiority (Kaye et al., 2018) Of note, no patients developed resistance. When data from phase III cUTI studies was used to investigate the probability of target attainment of the 4 g IV q8h as a 3 h infusion dose of meropenem-vaborbactam, the pre-clinical, free-drug plasma meropenem %T > MIC target ≥45% was achieved by 96.6 and 98.7% of all patients with cUTI with Enterobacterales (Wenzler and Scoble, 2020) Prior to TANGO II, which studied meropenem/vaborbactam versus best-available therapy in patients with carbapenem-resistant Enterobacteriaceae infections including HABP, VABP, cUTI, AP, and complicated intra-abdominal infections (cIAI), the plasma and intrapulmonary PK was evaluated in a single-center phase 1 study of 25 healthy volunteers after receiving meropenem/vaborbactam 4 g IV q8h over 3-h infusion for 3 total doses (Wenzler et al., 2015; Wunderink et al., 2018) Blood and bronchoalveolar lavage sampling were used to collect plasma and pulmonary samples to determine the concentration of meropenem and vaborbactam at various time points. The pharmacokinetic parameters of meropenem and vaborbactam in the plasma was as follows: C_max_ 58.2 ± 10.8 μg/ml and 59.0 ± 8.4 μg/ml, AUC_0-8_ 186 ± 33.6 μg h/mL and 204 ± 34.6 μg h/mL, V_ss_ 16.3 ± 2.6 L and 17.6 ± 2.6 L, CL 11.1 ± 2.1 L/h and 10.1 ± 1.9 L/h, and t_1/2_ 1.03 ± 0.15 h and 1.27 ± 0.21 h. The mean concentrations of meropenem in the plasma and epithelial lining fluid (ELF) ranged from 1.36 to 41.2 μg/ml and from 2.51 to 28.3 μg/ml, respectively. The mean concentrations of vaborbactam in the plasma and ELF ranged from 2.74 to 51.1 μg/ml and from 2.61 to 26.1 μg/ml, respectively. The ratios of ELF to plasma concentrations based on the mean AUC_0-8_ values were 0.63 for meropenem and 0.52 for vaborbactam. Overall, concentration-time profiles in the ELF of meropenem and vaborbactam were nearly identical and concentrations remained consistently several fold higher than the MIC_90_ of KPC-producing *Klebsiella pneumoniae* (Wenzler et al., 2015) Thus, patients in TANGO II received monotherapy with meropenem-vaborbactam at a dose of 4 g IV q8h over 3-h infusion. TANGO II was stopped early due to a significant reduction in mortality in patients using meropenem-vaborbactam (Wunderink et al., 2018) TANGO II was also the largest trial conducted in patients with CRE published thus far and the first and one of the only to use monotherapy. Together, the purposeful and well-planned drug development program for meropenem-vaborbactam lead to the development of an agent with very potent antibacterial activity and optimized PK-guided dosing which then demonstrated clinical success and garnered regulatory approval (Jorda and Zeitlinger, 2020).

### Eravacycline

Eravacycline differs from other tetracyclines based on modifications at C-7 and C-9 on the phenyl ring that confer increased activity and stability against tetracycline-specific efflux and resistance due to ribosomal protection proteins (Zhanel et al., 2016) It has broad-spectrum *in vitro* activity against a variety of aerobic and anaerobic Gram-negative and Gram-positive bacteria, including important pathogens that cause intra-abdominal infections with MIC90 values ranging from 1–4 mg/l (European Medicines Agency, 2018) It has poor activity against *Pseudomonas aeruginosa*, with MIC90 values of 16–32 mg/l, which is similar to other antibiotics in the class. An *in vitro* dynamic chemostat system was used to determine that free drug AUC_0-24_:MIC ratio was the PK/PD index most associated with decrease in bacterial density against *Escherichia coli* specifically. Across all five *E. coli* isolates included the magnitude of eravacycline free-drug AUC_0-24_:MIC ratio associated with net bacterial stasis and 1- and 2-log1- CFU reductions from baseline was 15.3, 20.5, and 28.8, respectively. An *in vivo* immunocompetent mouse thigh model was used to assess the mean free AUC/MIC magnitudes required for a net static response and a 1-log_10_ reduction in Enterobacteriaceae isolates and were found to be 2.9 ± 3.1 and 5.6 ± 5.0. The proposed dosing regimen for cIAI of 1.0 mg/kg q12 h was chosen based on the results of population PK models and non-clinical PK/PD targets for efficacy, *in vitro* surveillance data, and Monte Carlo simulations. Of note, in phase I studies using IV eravacycline, two regimens were initially selected for phase II studies: 1.0 mg/kg q12h and 1.5 mg/kg q24 h. The 1.0 mg/kg q12 h dosing regimen showed better tolerability compared to the higher dosing and was selected moving forward for phase II studies (European Medicines Agency, 2018). Additionally, phase II studies in cIAI that determined the likelihood of clinical success and target attainment noted that the average predicted percentage probability of clinical success was 98% across the MIC distribution for the 1 mg/kg q12 h regimen versus 97.3% with the 1.5 mg/kg q24 h regimen. The 1.0 mg/kg q12 h was chosen for phase III studies in cIAI because the success rates were numerically higher with this dose in addition to fewer noted adverse events (European Medicines Agency, 2018) Monte Carlo simulations were used utilizing the 1.0 mg/kg q12 h dosing regimen to evaluate PK/PD target attainment to further support its dosing in cIAI. Percentage probabilities of PK/PD target attainment were ≥90% at MICs of 0.015 and 0.12. At the MIC90 values of 0.5 and 1 ug/mL for Enterobacterales percentage probabilities of PK/PD target attainment equalled 0% (European Medicines Agency, 2018) IGNITE-1 was the phase III clinical trial that assessed the efficacy and safety of eravacycline versus ertapenem in patients with cIAI. This study found that eravacycline demonstrated noninferiority to ertapenem in this patient population. The microbiological responses for each patient in this study was later pooled together and no trend was noted towards a decrease in favorable response with increasing eravacycline MICs (Solomkin et al., 2017) IGNITE-2 studied the efficacy and safety of eravacycline compared with levofloxacin in cUTIs. Unfortunately, this study did not find noninferiority between patients receiving eravacycline and levofloxacin (TPHASE, 2018) There are some noted issues regarding this study and research leading up to this study. The dose utilized in this study was 1.5 mg/kg q24 h compared to a levofloxacin dose of 750 mg q24 h. Eravacycline tissue distribution is noted to be rapid, widespread, and concentrated in the trachea, adrenal gland, liver, aorta, and melanin-containing structures (European Medicines Agency, 2018) The majority of the drug is excreted via fecal or biliary elimination with a small fraction eliminated into the urine. When looked at in patients with renal dysfunction, it was noted that eravacycline exposure was similar in subjects with ESRD compared to those with normal renal function, demonstrating that renal dysfunction minimally effects eravacycline concentrations (European Medicines Agency, 2018) Eravacycline MICs were typically 2- to 8-fold higher in urine relative to broth for both *E. coli* and *K. pneumoniae* and only 2- to 4-fold higher when adjusted to the same pH. This increased with decreasing pH, demonstrating that pH has an impact on the *in vitro* activity of eravacycline (European Medicines Agency, 2018) Together this suggests that a more dedicated focus on understanding the PK of the agent and how different biologic matrices impact its PD may have led to an improved study design and potentially additional approvals.

## Summary

To combat the continued proliferation of antibiotic-resistant pathogens in the absence of rapid antimicrobial drug development, we must use our knowledge of antimicrobial PK and PD to maximize the efficacy, minimize the toxicity, and preserve the lifespan of our currently available agents. Pre-clinical PK/PD methods have improved dramatically over a short period of time and have now become integral and valued components of a robust drug discovery and development program. They have demonstrated the ability to de-risk an R and D program by ensuring that the dose used in Phase 3 trials is pharmacokinetically optimized to meet the PTA thresholds leading to success in clinical trials. Incorporating strong PK/PD principles from the beginning of the development program can lead to obvious success such as in meropenem-vaborbactam while a lack of appreciation for the intricacies of antimicrobial PK may have contributed to the lack of eravacycline success in cUTI.
